# Deficiency in the 15 kDa Selenoprotein Inhibits Human Colon Cancer Cell Growth

**DOI:** 10.3390/nu3090805

**Published:** 2011-09-05

**Authors:** Petra A. Tsuji, Salvador Naranjo-Suarez, Bradley A. Carlson, Ryuta Tobe, Min-Hyuk Yoo, Cindy D. Davis

**Affiliations:** 1 Cancer Prevention Fellowship Program, National Cancer Institute, Rockville, MD 20892, USA; 2 Molecular Biology of Selenium Section, Laboratory of Cancer Prevention, National Cancer Institute, Bethesda, MD 20892, USA; Email: naranjos@mail.nih.gov (S.N.-S.); carlsonb@mail.nih.gov (B.A.C.); tober@mail.nih.gov (R.T.); yoom@mail.nih.gov (M.-H.Y.); 3 Nutritional Science Research Group, National Cancer Institute, Rockville, MD 20892, USA; Email: davisci@mail.nih.gov; 4 Department of Biological Sciences, Towson University, Towson, MD 21252, USA

**Keywords:** HCT116 cells, HT29 cells, shRNA, selenium, cancer prevention

## Abstract

Selenium is an essential micronutrient for humans and animals, and is thought to provide protection against some forms of cancer. These protective effects appear to be mediated, at least in part, through selenium-containing proteins (selenoproteins). Recent studies in a mouse colon cancer cell line have shown that the 15 kDa selenoprotein (Sep15) may also play a role in promoting colon cancer. The current study investigated whether the effects of reversing the cancer phenotype observed when Sep15 was removed in mouse colon cancer cells, were recapitulated in HCT116 and HT29 human colorectal carcinoma cells. Targeted down-regulation of Sep15 using RNAi technology in these human colon cancer cell lines resulted in similarly decreased growth under anchorage-dependent and anchorage-independent conditions. However, the magnitude of reduction in cell growth was much less than in the mouse colon cancer cell line investigated previously. Furthermore, changes in cell cycle distribution were observed, indicating a delayed release of Sep15 deficient cells from the G_0_/G_1_ phase after synchronization. The potential mechanism by which human colon cancer cells lacking Sep15 revert their cancer phenotype will need to be explored further.

## 1. Introduction

Selenium is present in the environment, and distributed widely in the Earth’s crust in varying concentrations, and found in trace quantities in most plant and animal tissues. While not an essential nutrient for plants, it is an essential nutrient for humans and many other life forms [1,2]. Epidemiological, clinical and preclinical studies provide evidence that selenium may protect against colon cancer [3,4,5]. Part of the protective effect appears to be mediated through selenium containing proteins (selenoproteins). The 15 kDa selenoprotein gene (*SEP15*) is one of 25 known selenoprotein genes in humans and 24 genes in rodents [6]. Similar to many other selenoproteins, the gene product, Sep15, belongs to the class of thiol-disulfide oxidoreductase—like selenoproteins [7], and has been shown to exhibit redox activity [8,9]. However, its mechanistic properties continue to be elucidated. In humans, *SEP15* is located on chromosome 1p31, a locus commonly deleted or mutated in cancer [10], and human polymorphisms in this gene are thought to reflect differential susceptibility to cancer [11,12]. Other studies also suggest a role of Sep15 in cancer prevention [12,13].

Interestingly, more recent studies suggest that Sep15 may have an important role in promoting and/or sustaining colon cancer [14]. Mouse colon CT26 cells that were stably transfected with shRNA constructs targeting Sep15 displayed decreased growth abilities both under anchorage-dependent and anchorage-independent conditions. Moreover, the cells’ tumorigenic potential was decreased, as most mice injected with control cells had developed subcutaneous tumors, whereas few mice injected with Sep15-deficient cells developed tumors. The ability to form pulmonary metastases had similar results; *i.e*., mice injected intravenously with plasmid-transfected control cells developed more than 250 lung metastases per animal. However, mice injected with cells with down-regulation of Sep15 only formed 7.8 ± 5.4 metastases. This influence on cellular proliferation was likely mediated, at least in part, by the observed G_2_/M cell cycle arrest in mouse colon cancer cells that lacked Sep15 expression.

Expression of Sep15, which is regulated by dietary selenium, is found to be higher in liver, kidney, prostate and thryroid tissues [15,16], but can also be detected in other tissues, such as colon [17]. A recent study demonstrated that absence of Sep15 expression in mice *in vivo* does not reveal any strong phenotypes or gross abnormalities [16]. However, previous observations in cells [9,18,19] as well as these recent observations of mild oxidative stress in livers and cataract formation in lenses in Sep15 knockout mice [16] indicate a role of Sep15 in redox homeostasis as well as glycoprotein folding.

Knockout of Sep15 in mice *in vivo* has also been shown to influence colon cancer susceptibility [17]. The total number of carcinogen-induced aberrant crypt foci per colon and the number of aberrant crypts per focus were significantly lower in Sep15 knockout mice compared to wild type and heterozygous littermate controls. Because aberrant crypt foci serve as a surrogate biomarker for colon cancer risk in humans [20], these results indicate that, unlike previous observations in human mesothelioma cells [12], a lack of Sep15 expression may be protective against colon tumor formation *in vivo.*

The purpose of this study was to investigate whether the effects observed when Sep15 was removed in mouse colon cancer cells through RNAi technology, were recapitulated in human colon cancer cells, for which we utilized two commercially available, p53-expressing human colorectal carcinoma cell lines, HCT116 and HT29. Both cell lines expressed Sep15 in appreciable levels. Our results demonstrate that targeted down-regulation of Sep15 in these human colon cancer cell lines indeed resulted in similarly decreased growth under anchorage-dependent and anchorage-independent conditions. However, the magnitude of the decrease in cell growth was much lower than in the mouse colon cancer cell line investigated previously [14], and the potential mechanism will need to be explored further.

## 2. Experimental Section

### 2.1. Materials and Reagents

^75^Se (specific activity ~1000 Ci/mmol) was obtained from the Research Reactor Facility at the University of Missouri (Columbia, MO, USA). Dulbecco’s Minimum Essential Medium (DMEM), Gibco^®^ fetal bovine serum, puromycin, NuPage^®^ 4–12% polyacrylamide gels, LDS sample buffer, See-Blue Plus2 protein markers, and TRIzol^®^ reagent were purchased from Invitrogen (Carlsbad, CA, USA), iScript™ cDNA synthesis Kit from Bio-Rad Laboratories (Philadelphia, PA, USA), SYBR green supermix from New England Biolabs (Ipswich, MA, USA), primers for real-time PCR from Sigma-Genosys (St. Louis, MO, USA), Ribonuclease A from Sigma-Aldrich (St. Louis, MO, USA), and Noble agar from Becton, Dickinson and Company (BD, Franklin Lakes, NJ, USA). All other reagents used were commercially available and were of the highest quality available.

### 2.2. Culture of Mammalian Cells and Cell Growth Assays

HCT116 and HT29 cells were cultured in growth medium (DMEM supplemented with 10% heat-inactivated fetal bovine serum, 1% penicillin/streptomycin) in a humidified atmosphere with 5% CO_2_ at 37 °C. Both malignant cell lines were stably transfected with shSep15 or a control construct as described below. Cell growth was monitored by seeding cells in 6-well plates at 1 × 10^5^ cells/well in complete growth medium and counting cells for four days. 

### 2.3. Targeted Down-Regulation of Sep15

For the generation of HCT116 shSep15 cells, two Mission^®^ shRNA lentiviral transduction particles with validated sequences were chosen for targeting Sep15 knockdown (CCGGCTTTGCAGCTCTTGTGATCTTCTCGAGAAGATCACAAGAGCTGCAAAGTTTTTTG or CCGGGAAAGGAATGACAGCAGACTACTCGAGTAGTCTCGCTGTCATTCCTTTCTTTTTTG), and used as described by the manufacturer (Sigma-Aldrich, St. Louis, MO, USA). Briefly, cells were plated at 50% confluency, and viral particles were incubated overnight following addition of hexadimethrine bromide. Fresh medium was applied, and HCT116 Sep15 knockdown cells were selected by puromycin resistance. Both sequences resulted in very similar down-regulation of Sep15, and only results of one construct in comparison to shRNA lentiviral control particles are reported herein. 

For the generation of HT29 Sep15 knockdown cells, the first sequence was cloned in the pSuper retroviral plasmid (Oligoengine, Seattle, WA, USA) following the manufacturer’s instructions. GP-293 cells were transfected with 1.5 µg of the retroviral vector and 1 µg of an amphotropic envelope expression vector (pVSV-G). Medium was changed 24 h after transfection, and cell culture supernatants were harvested 48 h post-transfection, filtered through a 0.22 µm filter, and diluted (1:2) with fresh medium. Diluted viral supernatants containing 6 µg/mL hexadimethrine bromide were then added to HT29 cells (plated at a low confluency 24 h prior to the infection). HT29 Sep15 knockdown cells were selected by puromycin resistance.

### 2.4. Real-Time RT-PCR Analysis

Total RNA was extracted from HCT116 and HT29 cells using the TRIzol^®^ reagent. cDNA was synthesized using iScript™ with 1.5 μg of total RNA. For real-time quantitative RT-PCR, 1.5 μL of cDNA were used in 20 μL reactions by employing the MyiQ™ Single-Color Real-Time PCR detection system (BioRad Laboratories, Hercules, CA, USA). Specificity of the amplifications was verified through analysis of the melting point curves. The primers used for real-time PCR are shown in Table 1. The expression of mRNA levels was calculated relative to the expression of *GAPDH* as the internal control, and was graphed relative to expression in HCT116 control cells.

**Table 1:** Human real-time RT-PCR primers utilized.

Gene	Primer Sequence
*GAPDH*	5′-ACGTGTCAGTGGTGGACCTG—fwd
	5′-TGTCGCTGTTGAAGTCAGAG—rev
*GBP-1*	5′-CTATGAGGAACCGAGGAAGG—fwd
	5′-CACGTTCCACTTCAATCTCC—rev
*GPx1*	5′-GTGCAACCAGTTTGGGCATCS—fwd
	5′-CCGCAGGAAGGCGAAGAGAGG—rev
*GPx2*	5′-CCATCAACATTGAGCCTGAC—fwd
	5′-CTAAGGCTCCTCAGGACTGG—rev
*IFN- γ*	5′-TGTTACTGCCAGGACCCATA—fwd
	5′-TTCTGTCACTCTCCTCTTTCCA—rev
*SEP15*	5′-CCGGCGTTTGGGCTACGGTT—fwd
	5′-CCAGCTGAAGCAGGTTGAAC—rev
*TR1*	5′-CCATAGTTACTTTTGGCCAT—fwd
	5′-GCGCAGCTGCAAAGCCTTGT—rev

### 2.5. ^75^Se Labeling of Cells

Cells were seeded in a six-well plate (1 × 10^5^ cells/well), incubated for 18–24 h, and then labeled with 50 μCi/well of ^75^Se for 24 h before harvesting. Cells were suspended in 1× NuPage^®^ LDS sample buffer, sonicated and 75 μg protein were electrophoresed on a NuPage^®^ 4–12% polyacrylamide gel. The gel was stained with Coomassie Blue, dried, and exposed to a PhosphorImager screen (Molecular Dynamics, GE Healthcare, Piscataway, NJ, USA).

### 2.6. Soft Agar Assay

Anchorage-independent growth was assayed as described previously [14,21] with a total of 3000 cells of each stably transfected cell line suspended in 3 mL of 0.35% agar in complete DMEM and spread onto 60 mm dishes masked with a basal layer of 3 mL of 0.7% agar in the medium. Cells were incubated at 37 °C, 5% CO_2_ for 20 days, and complete growth medium was applied to the dishes every 3 to 4 days. The colonies were visualized by staining with ρ-iodonitrotetrazolium violet overnight, scanned and counted.

### 2.7. Cell Cycle Analysis

HT29 and HCT116 cells were grown in complete medium to 60% confluency, washed twice with PBS, and maintained in serum-free medium for 48 h to induce G_0_/G_1_ cell cycle synchronization. Cells were then growth stimulated with complete growth medium for 6, 12, 24 or 48 h (*n* = 6). Cells were then washed with PBS, trypsinized and suspended in PBS (1–2 × 10^7^ cells/mL) and kept on ice for 15 min. Ice-cold 70% ethanol was added gradually and cells were fixed overnight. Cells were centrifuged and resuspended in Ribonuclease A (100 units) and incubated at 37 °C for 20 min. The suspension was stained with propidium iodide in the dark at 4 °C overnight, filtered through a 50 micron mesh, and acquired with a FACScalibur™ (BD, Franklin Lakes, NJ, USA). The percent of cells in each phase of the cell cycle was analyzed by ModFit LT v.3.0 (Verity, Topsham, ME, USA).

### 2.8. Statistical Analyses

Data are presented as means ± SE and were analyzed by ANOVA or Student’s *t*-test using GraphPad Prism (v.4, LaJolla, CA, USA). Differences with *P* < 0.05 were considered significant. Levels of statistical significance were indicated as follows: * *P* < 0.05, ** *P* < 0.01, *** *P* < 0.001.

## 3. Results

**Figure 1:**
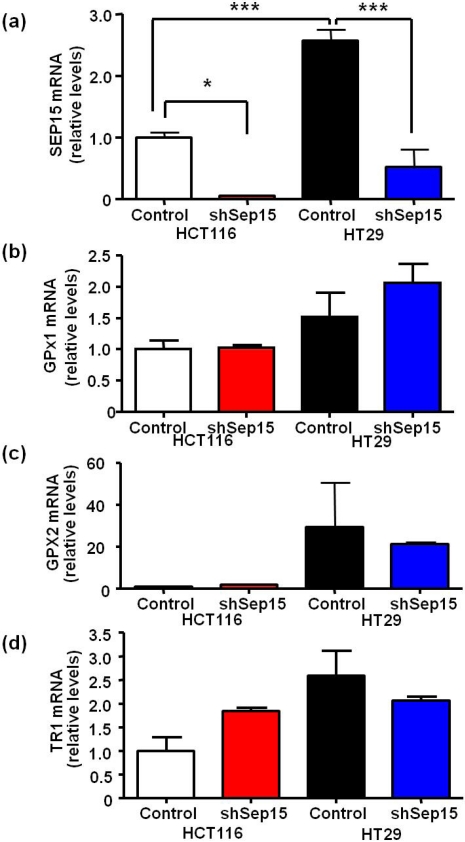
mRNA expression of selenoproteins in Sep15 deficient human colorectal cancer cells. mRNA levels of (**a**) Sep15, (**b**) GPx1, (**c**) GPx2 and (**d**) TR1 were measured using quantitative real-time RT-PCR, and *GAPDH* was used as an internal control. Data are displayed as means ± SE (ANOVA, *n* = 3) and were graphed relative to expression in HCT116 control cells (cycle threshold values were 17.6, 20.1, 19.6, 29.7 and 23.5 for *GAPDH*, *SEP15*, *GPX1*, *GPX2* and *TR1*, respectively).

**Figure 2:**
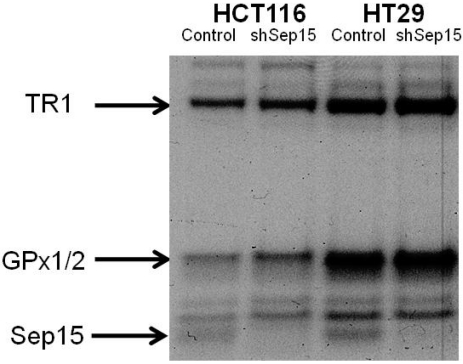
Protein expression of Sep15, GPx1/2 and TR1 upon targeted removal of Sep15 in HCT116 and HT29 human colorectal cancer cells, as determined by labeling cells with ^75^Se.

Using RNAi-technology, Sep15 mRNA expression was reduced significantly between 85% and 95% in both HCT116 (*P* < 0.05) and HT29 (*P* < 0.001) colon cancer cells compared to plasmid-transfected controls (Figure 1a), respectively. Interestingly, HT29 cells had an over two-fold higher Sep15 mRNA expression (*P* < 0.001) compared to HCT116 cells. Other selenoproteins, including glutathione peroxidases 1 and 2 (GPx1 and 2), and thioredoxin reductase 1 (TR1), did not exhibit statistically significant differences in mRNA expression (Figure 1b–d). Subsequently, expression of Sep15 and other selenoproteins was visualized by labeling cells with ^75^Se (Figure 2). Targeted down-regulation of *SEP15* resulted in loss of Sep15 protein in shSep15 cells compared to the plasmid-transfected control cells in both HCT116 and HT29 cells. The relatively higher expression of basal Sep15 mRNA in HT29 cells was recapitulated by the band strengths of the selenoproteins analyzed by ^75^Se labeling. This may indicate a higher production and/or turnover rate of Sep15 in these cells.

### 3.1. Cell Growth

**Figure 3:**
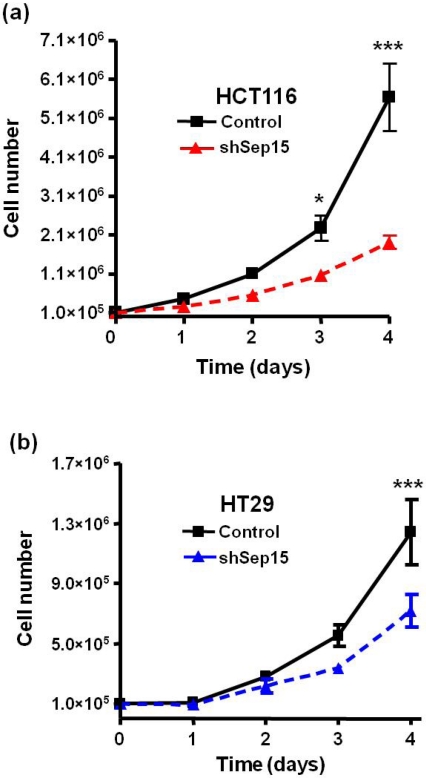
Effects of Sep15 deficiency on cell growth over a four day period with (**a**) HCT116 cells and (**b**) HT29 cells. Values are means ± SE (*t*-tests, *n* = 3–6).

HCT116 and HT29 human colon cancer cells that lack Sep15 exhibited a significantly reduced cell growth as early as three days after seeding (Figure 3). The growth rate was significantly reduced, and, in both the HCT116 and HT29 cell lines, there were 50% (*P* < 0.001) and 40% (*P* < 0.001) fewer shSep15 cells after four days compared to control cells, respectively (Figure 3a,b). 

### 3.2. Anchorage-Independent Growth in Soft Agar

**Figure 4:**
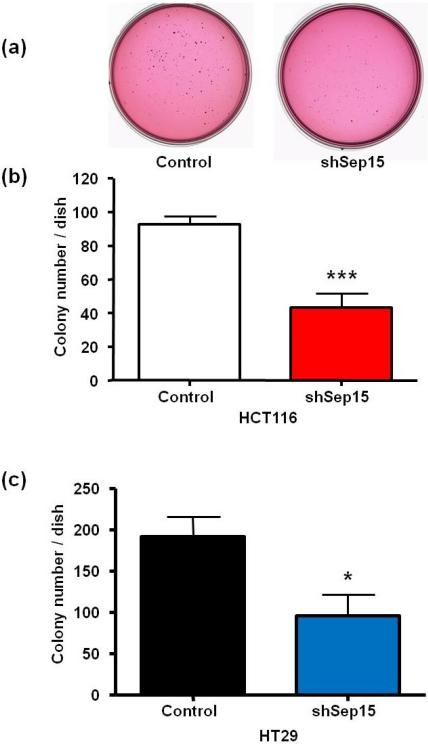
Effects of Sep15 deficiency on anchorage-independent colony formation in soft agar as quantitated by staining cell colonies with *p*-iodonitrotetrazolium overnight. (**a**) Representative soft agar dishes with stained colonies of HT29 cells after 20 days; numbers of colonies of (**b**) HCT116 cells and (**c**) HT29 cells. Values are mean ± SE (*t*-test, *n* = 4).

The ability to grow unanchored in soft agar, which is a characteristic of many cancer cells, was also evaluated for both cell lines (Figure 4a–c). A representative soft agar dish with stained HT29 cell colonies is shown in Figure 4a. In comparison to plasmid-transfected control cells, (Figure 4b) HCT116 and (Figure 4c) HT29 cells transfected with the shSep15 construct had a significantly reduced ability to grow anchorage-independently in soft agar (*P* < 0.001 and *P* < 0.05, respectively), compared to the plasmid-transfected control cells. Furthermore, for both cell lines, colonies in control dishes appeared generally larger than those observed in dishes with shSep15 cells.

### 3.3. Cell Cycle Analysis

**Figure 5:**
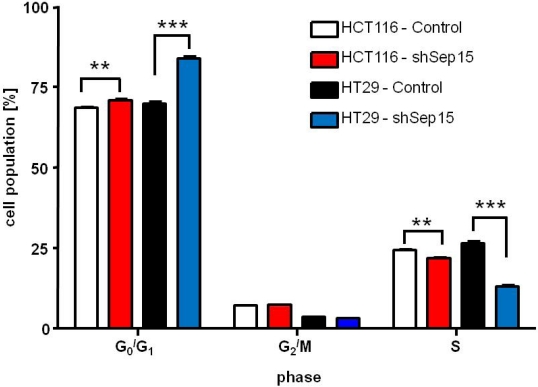
Cell cycle analysis of HCT116 and HT29 cells. Percent of cells in each phase of the cell cycle as determined by FACS analysis at six hours after release from synchronization with serum is shown. Values are means ± SE (*t*-tests, *n* = 6).

Fluorescence-activated cell sorting (FACS) was used to evaluate whether targeted down-regulation of Sep15 in human colon cancer cells would result in G_2_/M cell cycle arrest as suggested by our previous results with mouse colon cancer cell lines [14]. HCT116 shSep15 cells only exhibited a very small, but statistically significant larger percentage of cells in the G_0_/G_1_ phase compared to controls at six hours past cell cycle synchronization (*P* < 0.01, Figure 5). However, for HT29 shSep15 cells, a much more pronounced statistically significant delayed release of cells from the G_0_/G_1_ phase was observed six hours after synchronization (Figure 5). Other time points, *i.e.*, 12, 24 or 48 h following release from synchronization, did not demonstrate any marked differences between shSep15 cells and their respective controls (not shown).

### 3.4. Guanylate Binding Protein-1 and Interferon-γ Expression in Human Colon Cancer Cells

**Figure 6:**
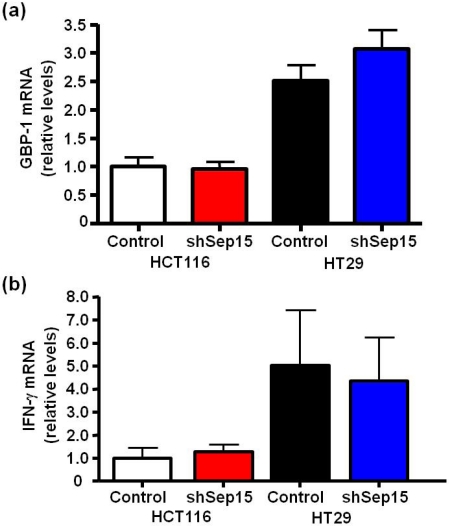
mRNA expression of guanylate binding protein-1 (GBP-1) and interferon-γ (IFN-γ). mRNA levels of (**a**) GBP-1 and (**b**) IFN-γin HCT116 and HT29 colon cancer cells were determined by quantitative real-time RT-PCR, with *GAPDH* used as internal control. Columns depict means ± SE (*t*-test, *n* = 6), and were graphed relative to expression in HCT116 control cells (cycle threshold values were 17.6, 22.3 and 31.5 for *GAPDH*, *GBP-1* and *IFN-γ*, respectively).

Because Sep15 deficiency in mouse colon cancer cells as well as in Sep15 knockout mice *in vivo* demonstrated a highly increased expression of GBP-1 compared to controls [14,17], mRNA expression of GBP-1 was also evaluated in human colon cancer cells. No statistically significant increase of GBP-1 mRNA was observed in shSep15 HCT116 or HT29 cells compared to their respective control cells (Figure 6a). Furthermore, mRNA expression of interferon-γ, a known inducer of GBP-1, was at the limit of detection and no significant differences were found between shSep15 knockdown cells and controls for either human cancer cell line (Figure 6b). The relatively higher levels of GBP-1 in HT29 cells corresponded to relatively higher interferon-γ levels compared to observations in HCT116 cells.

## 4. Discussion

Our previous study demonstrated that down-regulation of Sep15 may be protective against tumorigenesis and metastasis in murine colon cancer cells, and that these effects were mediated, at least in part, through an alteration of the cell cycle [14]. In the current study, we demonstrated that a targeted down-regulation of Sep15 in the two human colon cancer cell lines, HCT116 and HT29, also resulted in a reversal of the cancer phenotype. Both cell lines, upon down-regulation of Sep15, demonstrated significantly reduced growth abilities under both anchorage-dependent and -independent conditions compared to their respective controls. It appears that removing Sep15 expression may have protective effects against colon cancer in humans also. In contrast, as shown in our previous study, Sep15 deficiency had no effect on anchorage-dependent or -independent cell growth in mouse Lewis lung cancer cells [14]. It remains to be investigated, if this apparent tissue specificity of Sep15 observed previously in murine cancer cells will also apply to human cancer cells. Furthermore, the mechanism through which Sep15 acts in human colon cancer will need to be elucidated further. 

In mouse colon cancer CT26 cells, a clear G_2_/M phase arrest was observed in cell cycle analysis [14], whereas a delayed release from G_0_/G_1_ after cell cycle synchronization was observed in human cells. This delayed response was especially noticeable in HT29 cells, where shSep15 cells remained in the G_0_/G_1_ phase significantly longer than their corresponding control cells. This observation indicates that a loss of Sep15 expression in at least some colon cancer cells results in an alteration of the cell cycle.

We analyzed the mRNA expression of the interferon-γ inducible GBP-1 in both HCT116 and HT29 cells. Our previous results had indicated that targeted removal of Sep15 in mice *in vivo* also induced a very strong up-regulation of the large GTPase GBP-1 [17], which is thought to function as an anti-apoptotic protein [22]. This up-regulation of GBP-1, in absence of Sep15 expression, has also been found in the mouse colon cancer cell line CT26 [17]. Because up-regulation of GBP-1 expression in humans has been associated with longer survival in colon cancer patients [23], GBP-1 is considered to be an activation marker of endothelial cells during inflammatory diseases. Unlike in mouse colon cancer CT26 cells, no statistically significant increase of GBP-1 mRNA was observed in human colon cancer shSep15 cells compared to their respective controls. HT29 cells, which exhibited a two-fold higher GBP-1 expression than HCT116 cells, and a corresponding higher interferon-γ expression, showed a slight increase in GBP-1 mRNA expression (*P* > 0.05) upon targeted down-regulation of Sep15 compared to their controls. Future experiments should include human (colon) cancer cell lines with an even higher Sep15 expression than HT29 cells, which we would anticipate to have significantly increased GBP-1 levels as a response to loss of Sep15. 

## 5. Conclusions

Reduction of Sep15 resulted in reversal of the cancer phenotype in human colon cancer cells, with limited regulation through changes in cell cycle. The slight increase in GBP-1 mRNA in HT29 cells may reflect a stronger effect in response to loss of Sep15 in these cells. Future studies should include targeted down-regulation as well as over-expression of Sep15 in these and other colon cancer cell lines, which may be reflecting human polymorphic Sep15 status, and thus may help further elucidate the function of Sep15 in colon cancer in humans. Furthermore, because Sep15 expression depends on the selenium status, these results are important in regards to differential intake of, and response to, dietary selenium and potential cancer risk.
